# Epigenetic Regulation of Skin Cells in Natural Aging and Premature Aging Diseases

**DOI:** 10.3390/cells7120268

**Published:** 2018-12-12

**Authors:** Donata Orioli, Elena Dellambra

**Affiliations:** 1 Istituto di Genetica Molecolare CNR, Pavia; via Abbiategrasso 207, 27100 Pavia, Italy; 2 Molecular and Cell Biology Laboratory, Istituto Dermopatico dell’Immacolata, IDI-IRCCS, Via dei Monti di Creta 104, 00167 Rome, Italy

**Keywords:** skin, stem cells, epigenetic mechanisms, aging, progeroid syndromes, premature aging syndromes

## Abstract

Skin undergoes continuous renewal throughout an individual’s lifetime relying on stem cell functionality. However, a decline of the skin regenerative potential occurs with age. The accumulation of senescent cells over time probably reduces tissue regeneration and contributes to skin aging. Keratinocytes and dermal fibroblasts undergo senescence in response to several intrinsic or extrinsic stresses, including telomere shortening, overproduction of reactive oxygen species, diet, and sunlight exposure. Epigenetic mechanisms directly regulate skin homeostasis and regeneration, but they also mark cell senescence and the natural and pathological aging processes. Progeroid syndromes represent a group of clinical and genetically heterogeneous pathologies characterized by the accelerated aging of various tissues and organs, including skin. Skin cells from progeroid patients display molecular hallmarks that mimic those associated with naturally occurring aging. Thus, investigations on progeroid syndromes strongly contribute to disclose the causal mechanisms that underlie the aging process. In the present review, we discuss the role of epigenetic pathways in skin cell regulation during physiologic and premature aging.

## 1. Introduction

**Figure 1:**
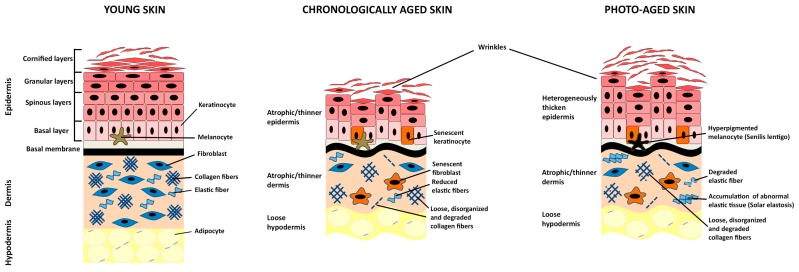
Morphological features of young, chronologically- and photo-aged skin. Schematic organization of the young, chronologically aged or photo-aged skin tissue. Human skin is constituted by three tissue layers: epidermis, dermis, and hypodermis. The epidermis is a stratified epithelium composed of keratinocytes organized into four major layers (basal, spinous, granular, and cornified layers) at progressive differentiation stages including melanocytes. The dermis is populated by fibroblasts embedded by the components of the extracellular matrix made of collagen, elastic fibers, glycoproteins, and proteoglycans. Fibroblasts are the main producers of the extracellular matrix components. The hypodermis is mainly populated by adipocytes. Morphological changes occurring with age are indicated in chronologically- and photo-aged skin.

The skin protects the body from environmental stresses, such as water loss and microorganism infection, and is made up of three tissue layers: the epidermis, the dermis, and the hypodermis (Figure 1) [1].

The human epidermis consists of four major cell layers composed of keratinocytes in stages of progressive differentiation [2]. Stem cells (SCs) and transient amplifying (TA)-cells are located in the basal layer and are instrumental for proper human epidermal regeneration. SCs are endowed with self-renewal capacity and persist during a lifetime. They contribute to both epidermal renewal and repair by continuously generating pools of TA-progenitors that persist for limited periods (3–4 months). Indeed, long-lived, slow cycling SCs generate activated SCs that enter into a transient state of rapid proliferation giving rise to TA-cells, which constitute the largest group of proliferating cells endowed with different proliferative capacities [2,3,4,5]. Following several divisions, TA-cells withdraw from the cell-cycle and generate post-mitotic keratinocytes that migrate upwards to compose suprabasal and upper layers by executing their terminal differentiation program. This process is tightly regulated by temporal and spatial gene expression modulation. The suprabasal layers are characterized by early differentiation markers (e.g., keratins 1 and 10), whereas the upper layers express late differentiation genes (e.g., loricrin, involucrin). The outermost layers are characterized by terminally differentiated and enucleated corneocytes that are continuously removed and replaced by cells from the strata below [2]. Thus, the clonal conversion from SC to TA- and thereafter, to post-mitotic cells, is a continuous process that maintains the integrity of the epidermal structure [3]. Epidermal homeostasis relies on a finely tuned processes that dictate the choice between SC self-renewal and differentiation [2]. Keratinocyte SC de-regulations may result in skin aging and/or tumorigenesis.

The dermis is a connective tissue populated by fibroblasts that are responsible for the synthesis and secretion of collagens and other matrix proteins (fibronectin, elastin, and glycans) into the extracellular environment. Matrix components maintain the skin architecture and confer elasticity as well as resistance and strength to tissue [1,6]. Human dermal fibroblasts are composed of heterogeneous subpopulations with distinct gene expression profiles according to the anatomical site of origin and different physiological and pathological conditions [7]. Notably, fibroblasts are implicated in almost every skin process by interacting with both the epidermal and the other resident dermal cells, such as endothelial, neural, adipocytes, and inflammatory cells. The signaling from the dermal compartment is fundamental for the maintenance and homeostasis of epidermal SCs. Moreover, dermal fibroblasts are involved in various physiopathological conditions including wound healing, fibrosis, aging, and skin cancer [1,7]. 

At last, the hypodermis is the subcutaneous adipose layer underneath the dermis that surrounds hair follicles and connects the skin to the below muscles and bones. Even though the activity of hypodermal adipocytes is relevant for skin homeostasis and the separation between the dermal and hypodermal layers is not always well defined [8], the following review will focus on the epigenetic changes and transcriptional regulatory networks associated to an age-dependent functional decline of epidermal SCs and dermal fibroblasts.

## 2. Key Regulators of Epidermal Homeostasis

Various signaling and transcriptional pathways regulate in a stage-specific manner the expression of genes implicated in epidermal SC homeostasis, proliferation, differentiation and aging. The clonal evolution of SCs is, in part, regulated by the tumor suppressor p16^INK4a^ and the transcription factor p63 [3,9]. They are modulated during clonal conversion accomplishment, and their over-expression or down-regulation impairs this process [10,11,12]. p16^INK4a^ is an inhibitor of Cdk4/cyclin D complex and maintains the retinoblastoma protein (pRb) in its hypophosphorylated active state, preventing the E2F-mediated transcription and blocking the entry of proliferating cells into S phase [13]. Instead of playing a primary role in the continuous epidermal regeneration [14], p16^INK4a^ is rather a master sensor of aberrant chromatin status that rapidly drives cell cycle exit and senescence [15]. Therefore, a key requirement for the maintenance and survival of the skin SC population throughout life is the repression of p16^INK4a^, thus, explaining the tight regulation in skin cells of p16^INK4a^ encoding gene (*CDKN2A*), which belongs to the *INK4a/ARF* locus [16].

Differently, p63 is a master regulator of epidermal morphogenesis. It acts as a transcription factor and is implicated in the maintenance of keratinocyte self-renewal and/or cell fate decision [17,18]. The *TP63* gene encodes several isoforms of p63 due to the presence of alternative promoters, different translation initiation sites, and alternative splicing events [19]. In human epidermis, ∆Np63 is the predominant isoform and plays a key role in keratinocyte proliferation and differentiation process through a Myc-regulated gene network and the interaction with several other transcription factors (AP-1, Klf4, Arnt, PPAR-alpha) [20,21]. Specifically, ∆Np63 and the protein encoded by its transcriptional target *REDD1* gene are essential for the proliferative capacity and differentiation of progenitor cells [22,23]. Furthermore, ΔNp63α promotes keratinocyte proliferation by suppressing the expression of senescence-inducing miRNAs [12]. Thus, the regulation of p63 expression is fundamental to skin regeneration.

Transcription factor-dependent and epigenetic regulatory mechanisms tightly collaborate to ensure proper epidermal homeostasis. Indeed, several epigenetic networks work in concert to preserve keratinocyte stemness and promote proliferation by repressing the transcription of the p16^INK4a^-encoding gene and other cell-cycle inhibitors as well as by inhibiting unscheduled activation of non-lineage- or terminal differentiation-associated genes. The unbalancing of opposite epigenetic enzymatic activities drives the transition from epidermal SC quiescence to activation. On the contrary, specific epigenetic networks may promote keratinocyte terminal differentiation by acting through the p63-regulated networks on epidermal differentiation complex (EDC) genes. In dermal fibroblasts, the epigenetic networks are involved in the repression of *INK4a/ARF* locus as well as inflammatory genes to fight against senescence and paracrine pro-inflammatory processes [9,24,25,26,27,28]. 

Finally, the deregulation of epigenetic pathways directing epidermal homeostasis can induce epigenomic instability and, in turn, skin aging.

## 3. Skin Aging

Aging is characterized by the accumulation of macromolecular damages, impaired tissue renewal, and progressive loss of physiological integrity. One of the hallmarks of aging is cellular senescence that is triggered by several intrinsic (e.g., telomere shortening, ROS overproduction) and extrinsic (e.g., UV radiations, nutrient deprivation, inflammation) stimuli leading to growth arrest and specific phenotypic alterations, such as chromatin and secretome changes. Cellular senescence prevents the uncontrolled proliferation of damaged cells and induces the clearance and the regeneration of the tissue. However, in old organisms, the accumulation of several damages and the deficiency of immunological surveillance result in senescent cell accumulation and impaired tissue homeostasis [29,30,31]. Studies in mouse models indicate a causative role of cellular senescence in driving in vivo aging. Indeed, the mediators of senescence may limit the long-term growth of self-renewing compartments, thus, prompting aging. p16^INK4a^ expression increases significantly with aging and the enhanced clearance of p16^INK4a^-positive senescent cells delays the onset of aging signs in progeroid mouse models [32,33]. Moreover, the deficiency of p63 in adult mice causes a cell growth arrest that impairs tissue regeneration and induces the appearance of aging features [34]. 

Skin aging can be distinguished in intrinsic or chronological aging and extrinsic or photo-aging, which are superimposed in the sun-exposed area of the body [35,36].

### 3.1. Chronological Skin Aging

Chronological skin aging results from the passage of time and is mainly influenced by genetic or metabolic factors. Aged skin exhibits epidermal thinning, fragility, wrinkle formation, and loss of elasticity [35,37]. Histological features are epidermal atrophy, reduced amounts of dermal fibroblasts and collagen fibers, which are loose, thin, and disorganized (Figure 1) [35,37].

The thinning of the epidermis depends on progressive keratinocyte SC dysfunctions and lower epidermal turnover, which are associated with the decline of skin barrier function and wound healing capacity [38]. Studies in mice and humans suggest that the reduced tissue regenerative capacity is not necessarily due to a decline in SC number or self-renewal but rather to a minor ability to produce progenitor, TA- and differentiated cells [39]. Nevertheless, the number of TA-cells increases in aged epidermis likely because they slow down the cell cycle compared to young TA-cells [16]. Moreover, during each replication cycle, telomeres become shorter and trigger a persistent activation of DNA damage response pathways thereby leading to cellular senescence [40]. p16^INK4a^ and p63 are mediators of keratinocyte senescence. Specifically, p16^INK4a^ is undetectable in keratinocytes from young donors and gets up-regulated during both replicative and premature senescence [10,11]. In contrast, p63 expression decreases during keratinocyte replicative senescence and its silencing are sufficient to induce cellular senescence [10,12]. Notably, p16^INK4a^ expression directly correlates with chronological aging of human skin in vivo. The number of p16^INK4a^-positive cells increases with age in both epidermal and dermal compartments [11,41]. Moreover, aged keratinocytes are characterized by reduced expression of p63 [11,12]. 

The age-dependent remodeling of the dermis is mainly due to the dysfunction of long-lasting resident fibroblast populations that undergo continuous damage accumulation [42]. Aged fibroblasts lose the ability to remodel and organize the extracellular matrix (ECM) reducing the overall synthesis and secretion of collagens and elastins. Moreover, aged fibroblasts may alter the epidermal homeostasis by paracrine mechanisms [43]. Senescent fibroblasts display the accumulation of distinct heterochromatin structures designated senescence-associated heterochromatin foci (SAHF) that are induced by p16^INK4a^/pRb pathway activation. SAHF are silent domains that co-localize with the epigenetic mark H3K9me3 and the heterochromatin protein HP1, which may induce a senescent cellular state by transcriptionally repressing the E2F target genes involved in cell proliferation [44]. Aged fibroblasts are characterized by the presence of nuclear γH2AX-positive foci representative of DNA double-strand breaks that increase exponentially with age. These DNA damage foci co-localize with either telomeric DNA, thus, indicating telomere dysfunction [45], or with PML nuclear bodies that sustain damage-induced senescence growth arrest and inflammatory cytokine secretion [46]. In vivo aged dermal fibroblasts display enhanced secretion of the cysteine-rich, angiogenic inducer protein 61 (CYR61 or CCN1) that stimulates the secretion of pro-inflammatory cytokines and matrix metalloproteinases (MMPs). In turn, CCN1 and MMPs contribute to the acquisition and maintenance of the senescent cell state by negatively regulating collagen homeostasis and increasing the degradation of collagen, respectively [47]. 

### 3.2. Photo-Aging

Skin is continuously exposed to environmental insults that may lead to extrinsic aging. Specifically, chronic exposure to sunlight results in phenotypic changes globally termed photo-aging. Photo-aged skin is characterized by epidermal thickness heterogeneity, accumulation of immune cells and pigmentation alterations associated to the formation of “senilis lentigines”. However, major alterations occur primarily within the dermis: Collagen fibers disorganization and partial degradation, which cause wrinkling of the skin, the disintegration of elastic fibers, and accumulation of abnormal elastic tissue that characterize “solar elastosis” (Figure 1) [35,48]. 

Sun exposed skin is affected by both UV-A (320–400 nm) and UV-B (290–320 nm) radiations. UV-A rays are less energetic but penetrate deeper into the dermal layer. They induce DNA, protein, and lipid damages as well as dermal remodeling alterations through the generation of reactive oxygen species (ROS). UV exposure at a sub-erythemal dose is a potent stimulator for producing and releasing skin–fibroblast-derived elastase that leads to drastic alterations of dermal structure and “solar elastosis”. Moreover, UV-A exposure increases the production of MMPs, in particular of MMP-1 that hydrolyzes the interstitial collagen leading to the disorganization and progressive degeneration of dermal ECM [49,50,51]. Chronic UV-A irradiation also affects the synthesis of other ECM molecules of the dermis. It inhibits hyaluronan synthesis by down-regulating the hyaluronic acid synthases (HAS)-1, -2 and -3, thus, altering the dermal proteoglycan composition [42]. Furthermore, aged fibroblasts increase melanogenic gene transcription leading to hyperpigmentation and appearance of “senilis lentigines” [52]. 

Differently from UV-A, UV-B radiation is mainly absorbed by the epidermis and directly induces DNA lesions, such as cyclobutane-pyrimidine dimers (CPDs) and 6-4 photoproducts (6-4PPs), in keratinocytes. DNA photolesions may result in the generation of DNA mutations leading to cell senescence, apoptosis or carcinogenesis. Moreover, UV-B radiation stimulates keratinocytes to release soluble factors such as cytokines, interleukin (IL)-1α and IL-6 that give rise to an inflammatory response, known as “sunburn”. Through a paracrine mechanism, keratinocytes also induce the secretion of MMP-1 from dermal fibroblasts [53,54]. It may be relevant to note that in vitro, human dermal fibroblasts are more susceptible to UV exposure than epidermal keratinocytes [55].

## 4. Epigenetic Changes in Skin Aging

Epigenetic regulatory networks consist of three major events: DNA modifications (mainly DNA methylation), histone modifications (mostly histone methylation or acetylation), and recruitment of higher-order chromatin remodelers [28]. DNA and histone modifications affect gene transcription by altering histone–DNA and histone–histone interactions and, thus, regulating the accessibility of transcription factors and components of the transcriptional machinery to the chromatin. Histone modifications, which occur mainly at the amino-terminal portion of histone tails, often act cooperatively and synergistically to either repress or activate transcription [24,26,28].

**Figure 2:**
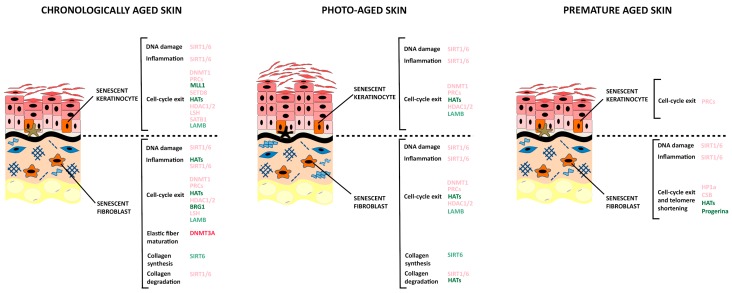
De-regulation of epigenetic modifiers affecting senescent epidermal keratinocytes and senescent dermal fibroblasts in chronologically-, photo- and premature-aged skin. Senescent cells are characterized by alterations of several cellular processes (DNA damage response, inflammation, and cell-cycle exit). Senescent fibroblasts display a modified metabolism of collagen and elastic fibers (elastic fiber maturation, collagen synthesis, and collagen degradation). Epigenetic modifiers inhibiting the cellular processes are indicated in light red when down-regulated and in dark red when up-regulated (or not down-regulated). Epigenetic modifiers activating the cellular processes are indicated in light green when down-regulated and in dark green when up-regulated (or not down-regulated).

Epigenetic changes are relevant for cellular senescence and aging (Figure 2). They can be modified by endogenous (e.g., intracellular signaling pathways) as well as exogenous stimuli deriving from lifestyle, diet, and environmental exposure (e.g., UV radiations, smoking, physical activity, antioxidant intake, caloric restriction) [56].

The chromatin of elderly subjects is characterized by histone loss, incorporation of different histone variants, altered DNA methylation and histone modification patterns, recruitment of different chromatin modifiers, and altered transcription profiles. Many studies indicate that epigenome modification contributes to the aging process [57]. Indeed, chromatin remodeling and histone post-translational modifications are critical for the recruitment and activation of DNA repair pathways and, therefore, for the maintenance of genomic integrity. The phosphorylation of histone H2A variant (γ-H2AX), the methylation on histone H3 lysine 79 (H3K79me), and the acetylation on histone H3 lysine 9 (H3K9) and lysine 14 (H3K14) have a key role in damage sensing and chromatin opening. On the contrary, the dephosphorylation of H2AX, acetylation or deacetylation of histone H3 and H4 are important for chromatin restoration after DNA-break repair [58]. The histone variant H2A.J, whose up-regulation promotes the expression of inflammatory genes, accumulates in an age-dependent manner in mouse and human skin as well as in irradiated mouse skin [59].

### 4.1. DNA Methylation

DNA methylation is an epigenetic mechanism that is mostly associated with transcriptional repression and is capable of regulating several aspects of gene expression (e.g., long-term gene silencing and genomic stability maintenance). The methyl group is placed by one of the three DNA methyltransferases (DNMT1, DNMT3A, and DNMT3B) on the fifth position of cytosine, predominantly at the CpG dinucleotide islands, to generate the 5-methyl-cytosine (5mC) base. DNMT1 is known as “maintenance DNMT” since it preserves the original methylation patterns during cell divisions. On the contrary, DNMT3A and DNMT3B are “de novo” DNMTs that catalyze new methylation marks. The presence of 5mC on the DNA mainly inhibits transcription by preventing the binding of specific transcription factors or by recruiting Methyl-CpG-binding proteins (MBPs) and histone deacetylases that, in turn, induce chromatin condensation. However, in some sporadic cases, DNA methylation contributes to gene activation: CpG methylation of the CRE sequence (*TGACGTCA*) enhances the DNA binding of the C/EBPα transcription factor, which regulates the expression of differentiation genes in several cell types [24,26,60,61].

A passive loss of 5mC marks can occur through several mechanisms, such as down-regulation of DNMT enzymes, cytosolic localization of DNMT, impairment of DNMT recruitment on DNA, decreased levels of the methyl donor S-adenosyl methionine, and inhibition of DNMT enzymatic activity [62]. The active loss of 5mC can be due to the Ten–Eleven Translocation (TET) family enzymes, which convert 5mC into 5-hydroxymethylcytosine (5hmC), and, in turn, into 5-formylcytosines and 5-carboxylcytosines. These modifications are substrates of the base excision repair pathway (BER) and are reverted to cytosine actively reducing the 5mC marks on DNA [63]. However, 5hmC is not only an intermediate of DNA demethylation, but rather a stable epigenetic mark associated with euchromatin [62].

DNA methylation is instrumental for suppressing the expression of genes involved in cell cycle exit and keratinocyte terminal differentiation [64,65,66]. Thus, the maintenance of the DNA methylation patterns is important to preserve skin progenitor cell identity and self-renewal whereas their remodeling after internal or environmental stimuli is associated with skin aging.

Young individuals display similar DNA methylation patterns (methylomes) whereas they become divergent in the elderly. These age-related changes in DNA methylation involve both hypermethylation and hypomethylation events and are associated with the progressive accumulation of epigenetic damage, known as “epigenetic drift” [67]. Studies carried out on monozygotic twins have shown that aging-associated DNA methylation changes are evident in monozygotic twins who lived apart for long periods, suggesting an environmental component of the “epigenetic drift” hypothesis [68,69]. Some of the age-related methylation changes that involve specific genomic regions are directional and not stochastic. Indeed, several studies indicate the presence of aging-associated differentially methylated regions (a-DMRs) that change over time in the same direction [62].

Comparing the methylomes between epidermal and dermal skin samples, a high degree of tissue specificity and inter-individual similarities within tissues have been found [70]. Methylation changes in the epidermis from young and old individuals are highly localized and display only minor quantitative differences demonstrating a limited destabilization of the epigenome during aging [71,72]. However, methylomes from aged skin samples are more heterogeneous than those from young ones, in support of the “epigenetic drift” hypothesis [70,72]. Discontinuous methylation changes can be, therefore, considered as features of aging methylome [72]. Interestingly, increased methylation heterogeneity has also been associated with fibroblast and epithelial cell senescence in culture [73,74,75]. Of note, there was a significant correlation between DNA methylation changes in in vitro long-term fibroblast cultures and in vivo aged fibroblasts, indicating that both processes might be regulated by similar epigenetic events [73]. Methylome data obtained from skin samples can be used to predict the chronological age of donors with elevated accuracy [72]. Intrinsic skin aging is mainly associated with widespread hypermethylation of CpG islands at gene regulatory elements [70,71,72]. Notably, hypermethylated DMRs have been associated with “bivalent” chromatin structures, which characterize genomic regions of transcription regulation in SCs. Aberrant DNA methylation at “bivalent” chromatin domain promoters is associated with a decreased capacity to differentiate in cell culture. Therefore, it is tempting to speculate that DMRs hypermethylation may induce a decline in the ability to produce progenitors, TA-, and differentiated cells [39,71]. Hypomethylated DNA sequences in aged skin are mainly co-associated with transcription enhancer elements in H3K9me3-marked regions [76], whereas, the indication of age-related global loss of DNA methylation as a major feature of intrinsic skin aging is still controversial [72,77]. The knowledge about DNA methylation changes might need a revaluation after the discovery of 5hmC because the conventional bisulfite sequencing method cannot discriminate between 5mC and 5hmC [78].

Age-related methylation changes have a minimal impact on in-cis gene expression, acting primarily to stabilize pre-existing baseline expression levels [71,72,79]. Analysis of gene co-expression networks allowed the identification of gene subsets whose expression changes in correlation with age and, in turn, with the loss of co-regulation by specific transcription factors. Skin samples from elderly display reduced connectivity of gene expression, suggesting that the age-related erosion of methylation patterns is paralleled by a reduced fine-tuning in transcriptional networks, probably mediated by methylation-dependent changes in transcription factor binding. Thus, the loss of epigenetic regulatory fidelity may be defined as a key feature of skin aging epigenome [72].

DNMTs can be involved in the age-related erosion of DNA methylation patterns. By repressing the *INK4a/ARF* locus, DNMT1 plays a key role in SC maintenance and tissue renewal, therefore, its dysfunction in keratinocytes and fibroblasts is instrumental for skin aging [64]. DNMT1 dysfunction is associated with epidermal cell senescence and impairment of skin homeostasis. The epidermal loss of DNMT1 in mice decreases hair follicle SC activation during aging and leads to progressive alopecia [64,80]. DMNT1 expression inversely correlates with p16^INK4a^ expression and chronological age in human skin samples as well as fibroblasts in culture [81]. Notably, DNMT1 knockdown significantly induces p21 up-regulation and premature senescence in human dermal fibroblasts [81,82]. UHRF1, a component of DNMT1 methylation machinery, is necessary for maintaining cell proliferation and it gets down-regulated upon stress-induced or replicative senescence of human keratinocytes as well as fibroblasts. Notably, decreased UHRF1 expression is a key initial event in the suppression of DNMT1-mediated DNA methylation [26,83]. *DNMT1* expression is also regulated by miR-377. A negative correlation has been identified between miR-377 and *DNMT1* expression in human skin tissues and dermal fibroblasts collected from UV-unexposed areas of differently aged individuals. By directly targeting *DNMT1*, miR-377 regulates the methylation of *TP53* promoter, and, therefore, induces the senescence of human skin fibroblasts [81]. Furthermore, aged fibroblasts display higher binding of DNMT3A on the *Lysyl oxidase-like 1* (*LOXL1*) promoter. *LOXL1* is an amino-oxidase enzyme involved in maturation of elastic dermal fibers, and its down-regulation is associated with the loss of skin elasticity [84].

The remodeling of DNA methylation patterns is also a feature of skin photo-aging. Besides DNA photolesions, UV irradiations induce oxidative stress, inflammatory responses, and immune suppression, which all can influence the epigenetic pattern. Specifically, sun exposure induces an epigenetic shift towards DNA hypomethylation in human skin and the degree of hypomethylation correlates with clinical photo-aging measures [70,77]. Among other events, a reduced expression of *DNMT1*, associated with the up-regulation of miR-377, is observed in photo-aged skin and UV-A-induced senescent human fibroblasts [81]. Indeed, it has been observed that in human dermal fibroblasts UV-A radiations reduce *DNMT1* expression through a ROS-mediated ZEB1 down-regulation [85]. In contrast, UV-B irradiations did not directly induce detectable changes of DNA methylation in human primary and immortalized HaCat keratinocytes [86,87]. However, the expression of TET enzymes (mainly TET2) and 5hmC levels significantly increase after UV-B irradiation [87].

### 4.2. Histone Methylation

Histone methyltransferases (HMTs) and demethylases (HDMs) covalently modify histones on their amino-terminal tails, altering the local chromatin environment and, in turn, gene transcription. Depending upon which residue of the histone tail is modified, histone methylation may promote transcription repression or activation. The trimethylation of histone 3 at lysine 27 (H3K27me3) and at lysine 9 (H3K9me3) are associated to facultative and constitutive heterochromatin, respectively, and, therefore, to gene silencing. In contrast, the trimethylation of histone 3 at lysine 4 (H3K4me3) and at lysine 36 (H3K36me3) are linked to euchromatin and transcriptional activation [26,88,89,90].

Among the methylated histone modifications, H3K27me3 is the most well-studied at the level of skin homeostasis. The presence of this transcriptional repressor epigenetic mark depends on the opposite activities of two families of chromatin modifiers: the Polycomb Repressor Complex (PRC) enzymes, which own methyltransferase activity and use it to catalyze histone methylation, and the histone demethylase Jumanji proteins that remove the methyl group [26,88,89].

The PRC complex activity involves two sub-complexes: PRC1 and PRC2. PRC2 is the initiator of transcription repression whereas PRC1 is a repressor maintenance complex. The PRC2 complex consists of the EED, Suz12, RBAp46/48, and methyltransferase EZH1/2 subunits that catalyze H3K27me3. This mark is necessary for the binding of PRC1 to the chromatin [88]. Canonical PRC1 is made of four different ortholog proteins, CBX, PCGF, HPH, and the E3-ligase protein (RING) that acts by catalyzing the monoubiquitylation of histone H2A (H2AK119ub1). This last histone modification prevents the RNA polymerase II transcription elongation by inducing chromatin condensation and gene silencing [89]. Through its chromodomain, the CBX subunit recognizes H3K27me3 and allows the recruitment of PRC1 to the target genes. Notably, CBX confers specificity to the complex as the different CBX proteins have a distinct pattern of chromatin binding [26,88,89]. The non-canonical PRC1 (ncPRC1) complexes are characterized by the presence of Rybp/Yaf2 and the lack of CBX components, and, therefore, their recruitment to DNA is independent of H3K27me3 mark [91]. Although the potential mechanism by which the ncPRCs mediate gene repression is largely unknown, these complexes are involved in embryonic development and stem cell maintenance [92,93,94].

PRC proteins play a key role in controlling epidermal SC identity and self-renewal by preventing the unscheduled induction of non-lineage, differentiation, and senescence genes [26]. On the contrary, the histone demethylase JMJD3 regulates the epidermal differentiation [95]. Reduced expression of PRC subunits is associated with skin aging as well as keratinocyte and fibroblast senescence both in vivo and in vitro models [11,41]. In particular, the levels of PRC2 in basal epidermal progenitors and fibroblasts decrease with age. EZH2 reduction leads to decreased levels of H3K27me3, disassociation of PRC1 complex from the chromatin and increased transcription of *INK4a/ARF* genes [25,96]. One of the most studied PRC1 subunits in human skin is the PCGF4 subunit/Bmi-1. Human keratinocytes and fibroblasts from aged donors are characterized by Bmi-1 down-regulation and early p16^INK4a^ expression, which are also correlated with SC depletion [11,96]. In agreement, Bmi-1 deficiency in mice leads to tissue atrophy and accelerated aging [97,98]. Notably, Bmi-1 is able to revert the aged phenotype modulating both p16^INK4a^ levels and clonal conversion in primary human keratinocytes from elderly donors [11]. In human dermal fibroblasts, miR-141-mediated Bmi-1 down-regulation induces cell senescence via p16^INK4a^ up-regulation [96]. The PRC-mediated transcription repression is counteracted by the action of Trithorax (TrxG) H3K4 histone methyltransferase complexes, which activate gene transcription through the deposition of H3K4me3 marks at H3K27me3 labelled promoters [99,100]. Specifically, the enzyme Histone-lysine N-methyltransferase 2A (KMT2A) or myeloid/lymphoid or mixed-lineage leukemia 1 (MLL1) activate the transcription of the p16^INK4a^-encoding gene during replicative and oncogene-induced senescence [101]. Therefore, the decline of PRC complexes may induce p16^INK4a^ expression by favoring the accessibility of p16^INK4a^-encoding gene to MLL1.

PRC complexes seem to have an active role also in photo-aging. Indeed, UV-A radiations modulate the expression of *EZH2* and *Bmi-1*, induce the senescence of human dermal fibroblasts and reduce hyaluronic acid (HA) synthesis. The use of GSK126, an inhibitor of the EZH2-methyl transferase activity, restores dermal fibroblasts growth after UV-A irradiation by modulating the expression of enzymes involved in HA synthesis and inhibiting the transcription of various photo-aging-related genes (e.g., *Smad2*, *Smad4*, *MMP-1*) [102,103]. Moreover, UV-B radiations induce human dermal fibroblast senescence through miR-101 induction and EZH2 reduction [104]. Globally, UV irradiation results in increased levels of the active H3K4me3 mark and decreased amounts of the repressive H3K9me2 mark as well as a concomitant increase of MMP-1 and MMP-3 secretion by human dermal fibroblasts [105].

Finally, the histone methyltransferase SETD8 (KMT5A) catalyzes the monomethylation of histone H4 at lysine 20 (H4K20me1), which represents a mark of gene activation. *SETD8* is a Myc-target gene that implicated in epidermal homeostasis and adult human keratinocyte self-renewal. The loss of Setd8 in mice results in loss of p63 and increased levels of p53 proteins [106]. In human epithelial cells, *SETD8* is down-regulated both in oncogene-induced and replicative senescence. Moreover, the inhibition of SETD8 is sufficient to trigger cell senescence [107].

### 4.3. Histone Acetylation

Histone acetyltransferases (HATs) and histone deacetylases (HDACs) modify histone tails by displaying opposite activities. HATs catalyze the acetylation of ε-amino groups of lysine residues promoting chromatin relax and subsequent gene transactivation. HDACs remove the acetyl groups and allow chromatin structure compaction and gene silencing. HATs are broadly categorized into three families: p300/CBP, Gcn5-related N-acetyltransferases (GNAT), and MYST [90].

p300 (also named EP300, E1A-binding protein p300) and CBP are closely related co-activators that acetylate multiple lysines on histone H3 (K14, K18, and K23) and on histone H4 (K5, K8, and K12). p300 and CBP enzymes play several roles in skin homeostasis: They control cell growth and early differentiation in keratinocytes through the stabilization of p63 and the p53-mediated regulation of *p21Waf1/CIP1* expression [108,109], they modulate p16^INK4a^ expression [110,111], maintain the integrity of skin barrier [112], and regulate the expression of pro-inflammatory and ECM genes in fibroblasts [90,113]. 

HDAC enzymes are classified in four classes: Class I, including HDACs 1–3 and 8; Class II, including HDACs 4–7, 9, and 10; Class III or Sirtuins (Sirt1-7), and Class IV, containing HDAC11 [90]. Whereas Class I HDACs play important roles in maintaining skin homeostasis, Class II is mainly involved in the wound healing process [28]. Among Class I, HDAC1 and HDAC2 are required for ΔNp63-mediated repression of the *INK4a/ARF* locus and, therefore, play an important role in epidermal SC self-renewal maintenance [114]. 

Class III HDACs or Sirtuins (Sirts 1–7) are NAD+ dependent enzymes relevant for several skin cell functions and processes, including control of energy metabolism and oxidative stress, cell survival, UV damage response, DNA repair, tissue regeneration and inflammation [115]. Specifically, SIRTs 1, 6, and 7 play a key role in epigenetic regulation and transcription facilitation. SIRT1 deacetylates histones H1 (H1K26), H3 (H3K9), and H4 (H4K16) and regulates the acetylation status of several transcription factors (e.g., nuclear factor-κB (NF-κB)), DNA repair proteins (e.g., poly-ADP-ribose polymerase 1 (PARP1) and NER factors), and the Werner syndrome ATP-dependent helicase (WRN). SIRT1 works in concert with p63 to regulate keratinocyte proliferation and the maintenance of epidermal progenitor cells by inhibiting cell senescence [21]. Moreover, SIRT1 inhibits collagen degradation suppressing the IL-1β-mediated induction of MMPs [116]. SIRT6 deacetylates the histone H3 (H3K9 and H3K56) and PARP1 to promote DNA repair. Specifically, SIRT6 may regulate DNA repair pathways, NFkB activity, and α-1-type 1 collagen (COL1A1) production in human dermal fibroblasts [117,118,119].

The modulation of HDACs and HATs enzyme activity is strongly implicated in skin cell senescence. The epigenetic regulator ING1 is part of both HAT and HDAC complexes and mediates chromatin remodeling and gene expression modification through the binding to H3K4m3. *ING1* gene encodes two major splicing isoforms: p33ING1b that induces apoptosis and p47ING1a that induces senescence. Although both INGl-isoforms have been found associated with HDAC complexes, INGlb exhibits a binding preference for the p300/CBP HAT complexes [120]. ING1a expression and ING1a-associated HDAC1 activity increase during fibroblast replicative senescence whereas ING1b expression decreases. Moreover, in primary human fibroblasts and HaCat cells, the overexpression of ING1a induces multiple markers of senescence, including SAHF containing HP1γ, flat morphology with large nuclei, inhibition of PCNA expression, and cell cycle arrest in the G1 phase, which strongly correlate with the activation of p16^INK4a^/Rb pathway [57,121]. Loss of function of both HDAC1 and HDAC2 determines p21 and p16^INK4a^ upregulation and extinguishment of SC proliferation [114].

Age-dependent changes of histone acetylation are coupled to the decline of metabolic activities and may influence global gene expression, which, in turn, affects health and longevity. SIRT1 and 6 seems to act as metabolic sensors regulating both transcription and genome stability depending on changes in the NAD^+^/NADH ratio [115]. Histone acetylation patterns are susceptible to quantitative alterations of key metabolites such NAD^+^ as well as mitochondrial metabolic activity, thus, allowing chromatin to function as a sensor of cellular metabolism [122]. Although mice moderately overexpressing SIRT1 do not live longer under standard diet conditions, they exhibit healthy aging features, such as improved glucose homeostasis, reduced tumor incidence, and reduced levels of the aging molecular marker, p16^INK4a^ or DNA damage [123]. Moreover, SIRT6-deficient mice display numerous progeroid and aging-like phenotypes [124]. 

Oxidative stress increases with age in human skin and leads to a PARP-mediated decline of NAD^+^ levels. A significant concomitant decrease of NAD^+^ levels and SIRT1 activity has been found in human non-sun exposed skin [125]. SIRT1 levels are consistently reduced during replicative senescence and epidermal aging. Moreover, SIRT1 silencing induces senescence similarly to p63 in keratinocytes. Specifically, ΔNp63 inhibits the expression of miR-138, miR-181a, and miR-181b that directly target SIRT1 [12]. SIRT1 also modulates H_2_O_2_-induced premature senescence in human primary keratinocytes. Although SIRT1 is not expressed in senescent keratinocytes, following forced expression its results are localized only in the cytosol suggesting a nuclear import dysfunction [126].

In addition, in human dermal fibroblasts, SIRT1 expression is significantly reduced with age [42,127]. Moreover, the expression of both SIRT1 and SIRT6 decreases in human dermal fibroblasts during replicative senescence in parallel with the appearance of aging biomarkers [128]. Notably, SIRT1 up-regulation or down-regulation results in delayed or accelerated fibroblast senescence, respectively [129]. The SIRT1-promoted cell proliferation and antagonized cellular senescence in human diploid fibroblasts appears partially mediated by the activation of ERK/ S6K1 signaling pathway [130]. Furthermore, SIRT1 mediates the inhibition of *MMP* transcription and, therefore, the preservation of collagens in the ECM of the dermis. Treatment of human dermal fibroblasts with the SIRT1-activator Resveratrol inhibits *MMP-1* expression, whereas SIRT1 down-regulation results in increased levels of MMP-1 and -3 [116].

As previously mentioned, also SIRT6 is involved in cell senescence and aging as it modulates the accessibility of DNA repair proteins to chromatin, represses nuclear factor (NF)-κB promoter through the histone deacetylation and, in turn, inhibits NF-κB target genes expression. Thus, SIRT6 down-regulation in human dermal fibroblasts results in increased DNA damage and NF-κB activity [118,131]. It has been shown that the activity of the DNA repair pathway BER declines with the aging of keratinocytes [132] and fibroblasts [131]. Dysfunctional BER contributes to the accumulation of mutations and, therefore, impacts on the age-related tissue homeostasis impairment. Notably, SIRT6 overexpression rescues the decline of BER in aged fibroblasts [131]. Similar to SIRT1, SIRT6 is implicated in the synthesis or degradation of skin collagen. SIRT6 down-regulation in human dermal fibroblasts reduces *COLA1* transcription, induces NF-κB activation, and increases MMP-1 secretion, suggesting that the reduced expression of SIRT6 in aged skin is directly implicated to the reduced levels of collagen fibrils [117].

Histone H3 hyperacetylation appears to be a relevant epigenetic mechanism in skin photo-aging. In comparison with non sun-exposed skin, solar irradiated skin samples exhibit a global increase acetylation of histone H3 in concomitance with increased p300 activity and reduced HDAC1 and SIRT1 expression. Gene promoters implicated in collagen degradation (*MMP-1*, *-3* and *-9*, and *Ahr* receptor), apoptosis (*PDCD5*), and inflammation (*ITIH5*) are hyperacetylated in the sun-exposed area of the skin [133]. In keeping with these data, UV irradiation induces histone H3K9/14 acetylation within the promoter regions of *ATF3*, *COX2*, *IL-8*, *MKP1*, and *MnSOD* genes in human keratinocytes [134]. Moreover, UV irradiation induces the acetylation of histone H3 by decreasing HDAC and increasing HAT activity in human dermal fibroblasts. This H3 hyperacetylation may be inhibited by p300 down-regulation, which plays a critical role in the transcriptional regulation of *MMP-1* upon UV exposure [135].

SIRTs play key roles in the cellular response to UV irradiation by reducing the oxidative stress, decreasing DNA damage signaling, and inhibiting *MMP* expression. Upon UV irradiation, SIRT1 deacetylates the NER factor XPA, thus, optimizing the activity of the NER repair pathway and, hopefully, protecting from photo-aging [136]. Photo-exposed skin displays increased levels of SIRT1 [137]. Moreover, SIRT1 confers protection against UVB-induced keratinocytes death via modulation of p53 and JNK [128,138]. Notably, it has been reported that UV radiation down-regulates SIRT1 expression through ROS-mediated JNK pathway activation in keratinocytes [138]. UV-B radiation down-regulates SIRT1 in a time- and dose-dependent manner in human immortalized HaCat keratinocytes. 

Furthermore, in human fibroblasts, UV-B radiation results in decreased SIRT1 protein levels [139] whereas SIRT1 overexpression protects dermal fibroblasts against UV-B-induced senescence. This protection is mediated by the suppression of p53 acetylation and, at the same time, by the deacetylation of the transcription factor FOXO3a that increases the cellular resistance to oxidative stress [140]. Resveratrol treatment inhibits *MMP-9* expression, thus, preventing collagen degradation in human fibroblasts or mouse skin after UV radiation exposure [141].

Differently, SIRT6 expression increases in human keratinocytes in response to UV-B exposure. Its silencing results in increased UV-B-induced apoptosis [142]. Moreover, UV-B induces the transcription of miR-378b that inhibits collagen I synthesis via SIRT6 in dermal fibroblasts [119].

Finally, in conjunction with the cellular senescence, SIRT4 expression increases in fibroblasts exposed to UV-A and UV-B radiation. Likewise, SIRT4 levels are higher in naturally photo-aged human skin samples [143,144]. 

### 4.4. Higher-Order Chromatin Remodeling and Three-Dimensional Genome or Ganization

The higher-order organization of chromatin and the spatial distribution of genes within the nuclear space, as well as the nuclear compartmentalization of chromatin-remodeling complexes and transcription machinery, play a key role in regulating gene expression and driving cell differentiation [24,145]. During epidermal regeneration and keratinocyte differentiation, the transcription factor p63 coordinates several chromatin-remodeling pathways by regulating the activity and expression of specific target genes. Among the p63-target genes are included proteins regulating covalent histone modifications (HDAC1/2), ATP-dependent chromatin remodeling factors (Brg1, Lsh/HELLS), higher-order chromatin remodeling proteins (SATB1,) and nuclear envelop assembly molecules (Lamins) [24,114,146,147,148,149].

Whereas the epigenetic mechanisms driven by DNMT1 or PRC complexes are conserved among different tissues and their de-regulation during aging results in self-renewal impairment of several SC types (i.e., hematopoietic, neural, epidermal SCs) [28,150], p63 is an epidermal master regulator and its age-related down-regulation specifically affect keratinocyte SC functions by its epigenetic targets [11,12].

ATP-dependent chromatin remodelers—Chromatin remodeling complexes play a direct role in gene expression regulation by coordinating DNA accessibility and dynamic of nucleosomes organization. Their activity depends on the hydrolysis of ATP as a source of energy [151]. 

The multisubunit SWI/SNF family complexes contain SWI2/SNF2-like ATPases (Brm/Smarca2/Snf2α or Brg1/Smarca4/Snf2β) that hydrolyze ATP to alter the histone-DNA interactions within nucleosome, leading to an open chromatin state and transcriptional activation. The SWI/SNF complexes also contain several regulatory components, called BAFs, endowed with different DNA target affinities and functions [151]. Brg1 is a p63 target gene, which is required for gene activation during epidermal barrier formation [152] and whose activity is prevented in epidermal SCs. Indeed, its subunit actin-like 6a (ACTL6a/BAF53a) inhibits the binding of the remodeler complex Brg1 to the gene promoter of *Krupper-like factor 4* (*KLF4*) transcription factor, whose function is to drive skin barrier formation [153,154]. Notably, the expression of *KLF4* decreases during chronological epidermal aging and keratinocyte replicative senescence. Specifically, *KLF4* silencing is sufficient to induce a senescent phenotype in primary keratinocytes [155]. Furthermore, Brg1 drives SAHF formation during senescence via its chromatin remodeling activity and/or by up-regulating p16^INK4a^ expression. Indeed, Brg1 may interact with pRB to drive SAHF formation [156]. SWI/SNF complexes are critical for the phosphorylation of H2AX and the formation of γ-H2AX foci following DNA damage. In particular, they facilitate DNA double-strand breaks (DSB) repair through the binding to γ-H2AX [157].

Lsh (HELLS) is a chromatin remodeler that belongs to the SNF2/helicase family, contains an ATP binding and a C-terminal helicase domains and is a meCpG stabilizer [158]. Lsh acts by recruiting DNMTs or through interaction with PRC1 complex and HDACs [159]. Furthermore, Lsh is a direct target of both p63 [147] and DNMT1 [160] that may cooperate to repress p16^INK4a^ expression and maintain a proliferative state in epidermal progenitors. Thus, p63 requires HDAC1/2 and Lsh/HELLS to repress the expression of anti-proliferative genes [114,147]. Notably, Lsh represses p16^INK4a^ expression by recruiting HDAC1 at its promoter and, through this event, it delays cell senescence in human fibroblasts [161]. Lsh transcription is activated by E2F1. Thus, E2F repression may be relevant for Lsh transcription decrease in senescent cells [162]. The loss of Lsh induces senescence in human keratinocytes and leads to accelerated skin aging in p63 heterozygous mice [12,147]. 

Genome organizers and nuclear architecture remodelers—CTCF is a zinc finger transcription factor that functions as genome organizer through the recruitment of cohesin, a protein essential for proper chromosome segregation, location, and gene expression. The genome organizer CTCF forms thousands of inter-chromosomal loops of different sizes and brings into close proximity to specific genes located on different chromosomes [163]. CTFC binds the *INK4/ARF* locus and inhibits its epigenetic silencing through chromatin remodeling. Thus, CTCF maintains high p16^INK4a^ levels and limits keratinocyte growth [164,165].

The specialized AT-rich binding protein 1 (SATB1) is a genome organizer that recruits chromatin remodelers at AT-rich sequences to regulate chromatin structure and gene expression [166]. SATB1 may activate or repress multiple genes depending on its posttranslational modifications and protein interactions. Indeed, SATB1 is a p63 target that allows the expression of EDC genes by remodeling chromatin architecture in the nucleus. However, SATB1 is also a direct target of miR-191 that induces keratinocyte senescence. Notably, *SATB1* expression decreases during keratinocytes replicative senescence, and its silencing induces a G1 block in cell cycle and an increment of senescence-associated markers [167]. Furthermore, SATB1 interacts also with pRB/E2F1 complex to repress the promoter of the p16^INK4a^ encoding gene [146,167,168]. 16^INK4a^ and the pRB/E2F pathway are critical for SATB1-induced cell cycle arrest [168]. 

The nuclear lamina is a protein structure that lines the inner membrane of the nuclear envelope (NE) and contributes to the size, shape, and mechanical stability of the nucleus. Besides its structural role, the nuclear lamina acts as an anchor for the peripheral elements of chromatin, thus, regulating the spatial organization of chromosome territories and the epigenetic state of the chromatin. Proteins of the nuclear lamina may be implicated in DNA replication, and DNA repair and transcription [169,170]. Notably, p63 regulates the nuclear shape and the expression of NE-associated genes, determining changes in heterochromatin organization and intranuclear position of keratin loci in keratinocytes [149]. The major structural elements of the nuclear lamina are the lamins [169]. Both A- and B-type lamins are required for chromatin organization and proper gene expression in skin cells [170,171]. During cellular senescence, the nuclear lamina undergoes dramatic remodeling [172,173]. Specifically, aberrant prelamin A isoform called progerin is present in cultured aged fibroblasts as well as in the dermis or few differentiated keratinocytes of elderly individuals, suggesting that senescence may be associated to the accumulation of an altered form of Lamin A [174]. Notably, overexpression of progerin in primary dermal fibroblasts results in reduced cell proliferation and premature senescence of a large number of cells [175]. Moreover, Lamin B1 levels decline during senescence, both in vitro cultures of human dermal fibroblasts and keratinocytes [172,176,177] and in vivo during the chronological aging of human skin [173,176]. Interestingly, loss of Lamin B1 is an early sensitive marker for the in vitro and in vivo quantification of UV-B induced keratinocyte senescence. It is worth noting, Lamin B1 levels are restored upon skin regeneration [177].

## 5. Epigenetic Regulation of Skin Premature Aging Diseases

### 5.1. Premature Aging Diseases

Progeroid syndromes (PSs) represent a group of clinical and genetically heterogeneous pathological entities characterized by an accelerated aging process affecting various (but not all) tissues and organs. This aspect earned them the name of segmental progeroid syndromes, and they feature many of the clinical and molecular hallmarks associated with the naturally occurring aging process. 

Because of these similarities, investigation on the PSs contributes to the understanding of the causal mechanisms that underlie aging. PSs do not show gender or ethnic preferences. Most of them appear in the first years of life and lead to life shortening. Major clinical features include short stature, alopecia, lipodystrophy, skin alterations, osteoporosis, and cardiovascular abnormalities [178]. Even though PSs display a large variety of clinical features and different propensities towards precious aging, the accumulation of DNA damage, increased genome instability, and epigenetic changes are clearly visible in all patient skins. Due to its easy accessibility, skin is becoming an ideal tissue in which to investigate the molecular processes responsible for aging or carcinogenesis. Even more, skin biopsies and primary skin cell cultures from patients affected by PSs represent a precious and irreplaceable source of biological material whose analysis is shedding light on the processes regulating senescence surveillance and progression [179,180].

According to the functional alteration responsible for the pathological symptoms, PSs can be classified as associated to DNA repair defects, to alterations of the nuclear envelope or telomere metabolism. This implies that the pathological mechanisms leading to premature aging are different, even though genomic instability is a common feature to all PSs.

#### 5.1.1. Progeroid Syndromes Resulting from DNA Repair Alterations

It is a consolidated notion that DNA repair defects lead to genome instability and to age-related pathological conditions, such as cancer, neurodegeneration, and decline of organ functionality. In humans, five genes encode the RNAQ helicases, a family of highly conserved proteins capable of hydrolyzing ATP to unwind double-stranded DNA during DNA replication, recombination, repair, and transcription [181]. Mutations in three of the *RECQL* genes, namely *RECQL2/WRN*, *RECQL3/BLM* and *RECQL4*, are responsible for the progeroid Werner (WS), Bloom (BS) and Rothmund–Thomson (RTS) syndromes, respectively. Concerning skin manifestations, all of them are associated with accelerated-aging features and cancer predisposition. In detail, WS patients reveal an increased tendency to melanoma formation, skin tightness, and ulcerations on ankles and elbows; BS is characterized by cancer proneness, UV-hypersensitivity leading to skin lesions, hypo and hyperpigmented area whereas RTS patients display poikiloderma, sparse grey hair, and increased susceptibility to cutaneous epithelial neoplasms, such as squamous and basal cell carcinomas [180].

Cocakyne (CS) syndrome, xeroderma pigmentosum (XP), and trichothiodystrophy (TTD) are caused by mutations in genes implicated in nucleotide excision repair (NER), the only pathway in human cells that removes the bulky lesions distorting the DNA double helix. Major targets of the NER pathway are the DNA photolesions (CPDs and 6-4PPs), caused by UV irradiation. Eleven different NER genes with different enzymatic activities are responsible for the XP, TTD, and CS clinical features, or the very rare cases showing a combination of XP and CS (XPCS) or XP and TTD (XPTTD) clinical features. XP is characterized by unusual skin photosensitivity, marked freckle-like pigmentation in the sun-exposed area, high skin cancer proneness and, in some cases, neurodegeneration, whereas CS and TTD are multisystemic disorders showing growth and mental delay, progressive functional deterioration, cutaneous photosensitivity but no skin cancer susceptibility despite the persistence of UV-induced DNA damage. In addition, TTD patients are characterized by brittle hair, and dry and scaly skin (ichthyosis) [182]. Accumulation of unrepaired DNA lesions in the epidermal stem or progenitor cells may explain the high skin cancer risk in XP patients. Indeed, the retroviral-mediated expression of wild-type *XPC* gene in primary keratinocytes from XP patients with mutations in *XPC* is, per se sufficient to restore the DNA repair capacity as well as to regenerate a fully differentiated epidermis [183]. Nevertheless, the same NER defects and mutation accumulation are observed in epidermal stem cells from TTD or CS patients, who do not develop skin cancer. The observation that many NER proteins are not only DNA repair factors but also key players in transcription provides a rationale to explain the distinct clinical features of NER disorders [182,184]. 

Mutations in 21 different genes (*FANC* genes) encoding proteins implicated in repairing interstrand cross-linked DNA give rise to Fanconi anaemia (FA) characterized by progressive bone marrow failure, short stature, skeletal and skin abnormalities, and high risk of hematopoietic and epithelial malignancies. Cells from FA patients are hypersensitive to DNA-cross-linking agents, which cause chromosomal breakage and genomic instability. Until recently, it was thought that all FA features were due to the accumulation of unrepaired DNA lesions in hematopoietic stem cells (HSCs). The discovery that FA proteins are involved in additional non-canonical biochemical functions, such as signaling pathways engaged in the cellular response to oxidative stress, viral infection or inflammation, has opened the possibility that additional failures contribute to FA pathogenesis [185,186].

The progeroid ataxia telangiectasia (AT) and Seckel syndrome (SS) result from mutations in genes encoding two related phospoinositol-3 kinases (PIKKs), the AT Mutated protein (ATM) and the AT and Rad3-related protein (ATR), respectively. Whereas the serine/threonine kinase activity of ATM is activated by the appearance of DSBs in the human genome, ATR responds to a wide range of lesions interfering with DNA replication [187,188]. Mutations leading to the inactivation of the ATM kinase result in early childhood ataxia (caused by progressive cerebellar neurodegeneration), telangiectasia (usually in the eyes), immune dysfunction, sterility, and cancer proneness. At the level of the skin, AT patients display pigmentary abnormalities and hair greying. Differently, loss of ATR function in SS patients is characterized by severe intrauterine growth retardation, neurodevelopmental abnormalities, dwarfism, and microcephaly. The cellular activation of either ATM or ATR leads to the phosphorylation of specific substrates acting as effectors of the DNA damage response (DDR) signaling pathway, which involves a broad spectrum of cellular processes relevant for genomic stability. Alterations of ATM effectors are observed in patients affected by other progeroid disorders: The AT-like disorder (ATLD) caused by mutations in the *hMRE11* gene and the Nijmegen breakage (NBN) syndrome due to mutations in *Nbs1* gene. Whereas ATLD appears as a partial phenocopy of the AT clinical features, NBS is characterized by growth retardation, microcephaly and cancer proneness. Notably, Mre11 and Nbs1 proteins are subunits of the same Mre11/Rad50/Nbs1protein complex [189,190].

Even though defects in the DNA repair pathways that account for the progressive aging features of PSs derive from different factors, they all flow into the common feature of chromosomal alterations and genomic instability. These events likely impair the expression of essential genes and result in metabolic dysfunctions, proteostasis failure, and impaired tissue homeostasis. Nevertheless, the molecular mechanisms linking DNA repair defects to aging is still elusive. 

#### 5.1.2. Progeroid Syndromes Associated to Alterations of the Nuclear Architecture

There is a group of segmental PSs that are generally referred to as laminopathies because they result from alterations in components of the nuclear envelope. The Hutchinson-Gilford progeria (HGPS), the atypical progeria (APS), the atypical Werner (a-WS) syndromes, and the mandibuloacral dysplasia with type A (MADA) are all caused by mutations in the *LMNA* gene. By alternative splicing the *LMNA* gene encodes lamins A and C proteins, which are intermediate filament components of the nuclear lamina [191]. Several post-translation modifications of the prelamin A polypeptide are required for the generation of a mature lamin A. This includes the farnesylation of the cysteine residue in the C-terminal domain CaaX of the protein, cleavage of the aaX peptide by the zinc metallopeptidase STE24 (ZMPSTE24), carboxyl-methylation of the farnesylated cysteine, and excision of the most 15 terminal residues by ZMPSTE24 [192]. In about 80% of HGPS patients, a mutation in *LMNA* gene causing the activation of a cryptic donor splice site results in a truncated isoform of prelamin A, known as progerin, which is farnesylated but not proteolytically cleaved by the metalloproteinase. The accumulation of progerin at the nuclear envelope is the primary cause of nuclear alterations in HGPS patients [193]. It is worth noting, progerin has also been found in some skin fibroblasts of young adults and in larger subsets of dermal fibroblasts, and in few terminally differentiated keratinocytes of older people [174]. Moreover, alterations of the lamin A maturation process also give rise to premature aging features as attested by the restrictive dermopathy (RD) and the mandibuloacral dysplasia with type B lipodystrophy (MADB), which arise from mutations in the *ZMPSTE24* gene. Finally, mutations in the *BANF1* gene give rise to the Néstor–Guillermo progeria syndrome (NGPS). The barrier to autointegration factor (BAF) encoded by the *BANF1* gene interacts with lamin A during the process of mitotic and post-mitotic nuclear assembly and was shown to influence the chromatin organization and gene expression regulation [194].

Besides the abnormal processing of lamin A, all progeroid laminopathies are characterized by altered nuclear shape (known as “nuclear bleb”) and persistence of DNA damage, which are associated with abnormal patterns of epigenetic modifications, altered recruitment of DNA repair proteins to the site of damage as well as unrepaired double-strand breaks and oxidative DNA lesions by intracellular ROS. The diversity of laminopathies is rather striking, even though they share common features (i.e., premature aging, axonal neuropathy, lipoatrophy, and myopathy). Skin alterations are observed in some of the LMNA-related disorders. In particular, hyperkeratosis of the epidermis, thinning of the dermis, and severe reduction of the elastic fibers are the cause of rigid and tight skin in RD patients. Moreover, the first signs of HGPS are skin alterations, including patches of hyper or hypopigmentation and sclerodermatitis, which are progressively associated with alopecia and skin wrinkling, reminiscent of the normal aging process [195]. 

Even though the nuclear envelop defects of laminopathies are not found in the PSs associated to DNA repair alterations, all these disorders share many clinical/cellular features, indicating that the molecular events causing the progeroid pathophysiological manifestations are still largely unknown. 

#### 5.1.3. PSs Associated to Alterations of Telomere Metabolism

The lack of the RNA-dependent DNA polymerase telomerase in most mammalian somatic cells results in the progressive shortening of telomeric sequences at the end of chromosomes and leads to a permanent cell cycle arrest, known as replicative senescence. Thus, attrition of telomeric sequence length represents an aging marker [196]. The progeroid disorders dyskeratosis congenita (DKC) and Hoyeraal–Hreidarsson syndrome (HHS) are monogenic disorders caused by mutations in genes encoding components of the telomerase complex. In particular, DKC is caused by defects in *TERC*, *TERT* or *WRAP53* genes that are engaged in telomere replication or by mutations in *CTC1* gene involved in telomere maintenance. HHS is caused by defects in *RTEL1* or *ACD* genes implicated in telomere-length regulation. Patients with DKC present skin defects, bone marrow failure, cancer predisposition, premature hair greying, and osteoporosis. Nowadays, HHS is considered as a severe form of DKC, which manifest early in life and is associated with additional symptoms usually not reported in DKC [197].

Notably, the lack of the WRN helicase in WS cells, as well as the accumulation of progerin in HGPS primary dermal fibroblasts, result in telomere attrition. This event can be counteracted by the ectopic expression of the catalytic subunit of the telomerase (hTERT), which results in HGPS immortalization, even if with a lower efficiency compared to the dermal fibroblasts from healthy donors [198]. Similar findings and an increased cellular lifespan were achieved by the hTERT retroviral transduction of human fibroblasts over-expressing progerin [175]. 

Therefore, investigations on PSs suggest a close relationship between DNA repair defects, nuclear envelope defects and telomere attrition. Even though triggered by different functional failure, physiological and premature aging share several molecular hallmarks among which are genomic instability, telomere shortening, loss of proteostasis, mitochondrial dysfunction, epigenetic alterations, deregulated nutrient sensing, stem cell exhaustion, cellular senescence and altered intercellular communication. 

### 5.2. Cellular Senescence in Premature Aging Diseases

The decline in DNA repair ability, the increase of oxidative stress, the shortening of the telomeres, the production of progerin or the loss of lamin B1 may drive cells towards senescence [199]. Similarly, skin cells from PS patients frequently show premature cellular senescence characterized by the expression of senescence-associated markers, such as loss of lamin B1, increased levels of p16^INK4a^, the presence of heterochromatin and γ-H2AX foci, senescence-associated secretory phenotype, impaired redox balance, and mitochondrial dysfunction [11,178,200,201]. 

Primary keratinocytes obtained from CS, TTD, and XPC patients exhibit senescent features in concomitance with high p16 and low p63 levels, similar to aged keratinocytes. These cells undergo a sudden clonal conversion that limits their life-span in culture [11,201]. Notably, the retroviral-mediated expression of wild-type *CSA* gene shows that CSA protein plays a relevant role in protecting keratinocytes from senescence by facilitating DNA damage processing, maintaining physiological redox status and keratinocyte clonogenic ability and reducing the senescence-associated secretory phenotype [85]. CS fibroblasts are characterized by increased levels of ROS, intense glycolytic metabolism and mitochondria abnormalities [202,203,204]. Moreover, these cells display an increased and persisting high levels of γ-H2AX foci, which co-localize with 53BP1 following infrared radiation (IR) exposure [200]. The loss of CSB in fibroblasts leads to activation of the p53/p21 pathway and expression of inflammatory genes [205]. Furthermore, primary dermal fibroblasts from TTD patients are characterized by the over-expression of MMP-1 leading to the partial loss of dermal interstitial collagens and wound healing defects, in agreement with the progeroid aging features of this disorder [206].

Fibroblasts from an HGPS mouse model present an up-regulation of p53-target genes. Notably, inhibition of p53 protein by the human papillomavirus E6 oncoprotein or the overexpression of hTERT can revert the proliferative defects associated with progerin expression [207,208]. The causative role of progerin in telomere shortening is still not fully elucidated. It has been observed that more than half of the cell telomeres localize to the nuclear lamina where they interact with the lamina-associated polypeptide-α (LAP2α). In HGPS cells the LAP2α interaction with telomeres is impaired, thus, suggesting a direct role of progerin in telomere structure and maintenance [209]. Indeed, the overexpression of wild-type lamin A leads to accelerated telomere loss and reduced lifespan [210]. Furthermore, progerin overexpression in human mesenchymal stem cells results in sporadic differentiation and premature exhaustion of the stem cell pool, thus, pointing to premature aging of the skin dermal layer and vasculature that relays on the Notch signaling pathways [211]. Similarly, the progeroid *Zmpste24*-knockout mouse model, which fails to process the lamin A protein, reveals an increased number of epidermal stem cells with a decreased proliferative activity in the hair follicle [212]. Overall, these finding suggest the idea that increased progerin levels in the skin of progeroid patients, as well as naturally aged individuals, may affect stem cell maintenance and directly impair skin regeneration. 

### 5.3. Epigenetic Changes in Premature Aging Diseases

Several lines of evidence support the idea that the molecular mechanisms leading to progeroid features are also implicated in the normal aging processes. Epigenetic changes affecting the status of the chromatin were shown to mark the age-related decline of progeroid cells (Figure 2) [29]. Indeed, chromatin disorganization is one of the major alterations associated to the impairment of prelamin A processing, as shown by cells from patients with HGPS and NGPS [213]. A large fraction (about 70%) of primary fibroblasts from HGPS patients show aberrant nuclear morphology, reduced levels of the heterochromatin protein HP1α, which acts as adaptor between the nuclear lamina and the chromatin, reduced levels of multiple members of the lamina-associated polypeptides (LAP2) as well as reduced levels of the heterochromatin-specific marker H3K9me3 [214]. A similar pattern of reductions is observed in about 61% of fibroblasts from normally aged individuals, with an age ranging between 81 and 96 years [208], strongly supporting the concept that progeroid disorders mimic the natural aging events. A global loss of H3K9me3 and changes in heterochromatin architecture have also been observed in human embryonic stem cells lacking the WRN protein functionality and induced to differentiate into mesoderm lineages. A similar decrease of heterochromatin marks and WRN protein are detected in the mesenchymal stem cells isolated from normally aged individuals [215].

Besides H3K9me3, progerin expression in HGPS cells affects the epigenetic control of facultative and constitutive heterochromatin, as shown by the reduced levels of H3K27me3 in the inactive X chromosome of HGPS females as well as by the up-regulation of H4K20me3, a marker for constitutive heterochromatin [216]. Both events have been found associated with natural cellular senescence [217,218].

Recent findings demonstrate an altered DNA replication timing in HGPS and RTS cells. The temporal order of genome duplication in cells is strictly associated with the chromatin organization and epigenetic marks, which are modulated during development. HGPS and RTS cells reveal a progeroid-specific signature of DNA replication timing, which is associated with the altered ratio of *TP63* isoform expression and resembling that of cellular senescence defects [219].

Altered epigenetic marks have also been observed in NER-defective cells [220]. Indeed, additional functions for the NER proteins XPC, XPA, RPA, XPG, and XPF have been demonstrated outside the DNA repair pathway. Even in the absence of a genotoxic attack, the recruitment of these NER factors at the promoter of active genes is necessary to achieve DNA methylation as well as histone post-translation modifications including methylation (H3K4 and H3K9) and acetylation (H3K9/14) [220,221]. Moreover, XPG and XPF are essential for the DNA demethylation and the formation of a CTCF-dependent chromatin looping between the promoter and the terminator of the active *RARβ2* gene [222].

Among the NER factors, the CS causative protein CSB is an SWI/SNF-like DNA-dependent ATPase that exhibits ATP-dependent chromatin remodeling activity in vitro. CSB disrupts the regulation of many genes involved in human aging, such as those genes affected by inhibitors of histone deacetylase and DNA methylation, as well as by defects in PARP-1 function and RNA polymerase II elongation [177]. Mouse and human CSB deficient cells display PARP-1 hyperactivation that leads to SIRT1 attenuation and, in turn, drives mitochondrial dysfunction and pro-inflammatory profile. PARP inhibition or NAD+ replenishment rescue the CS associated phenotypes in human cells and mice [176]. CSB modulates p53 activity and competes with p300 for p53 binding. The absence of CSB in patient fibroblasts induces the binding of p53 to p300 causing the stabilization of p53 and the activation of its target genes [179]. 

Moreover, an active heterochromatinization process following UV irradiation has been observed in NER-defective cells from progeroid patients presenting a combination of XP and CS clinical features. The heterochromatin alterations in these cells are due to the action of SIRT1 and results in wide transcription repression [178]. Since the generation of mouse models deficient for or over-expressing specific genes of interests, it has become well accepted that the SIRT family protects from age-related features and extends healthy life. Mice deficient for SIRT6 exhibit progeroid symptoms. Moreover, SIRT6 prevents telomere dysfunction by deacetylating H3K9 at the telomeric loci of human cells. Because SIRT6 is a chromatin-associated protein that promotes resistance to DNA damage and suppresses genomic instability in mouse cells, these findings link once more DNA repair pathways to the aging processes [116]. Nevertheless, SIRT6 also interacts with the stress-responsive transcription factor NF-κB and regulates the expression of some dependent NF-κB target genes, opening the possibility that the role of SIRT6 in aging may be ascribed to its ability to regulate the expression of aging-related genes including those implicated in cell survival, senescence, inflammation and immunity [223]. 

Primary keratinocytes obtained from TTD and XPC patients display Bmi-1 downregulation in concomitance with early expression of p16^INK4a^ and SC senescence. Notably, Bmi-1 expression restores keratinocyte clonogenic potential and delays cell senescence by modulating p16^INK4a^ transcription [11].

Finally, evidence for heterochromatin instability has also been reported in cells from patients affected by FA [224]. Even though epigenetic changes have not yet been identified in all progeroid syndromes, it is plausible to hypothesize that chromatin alteration associated with epigenetic and transcriptional deregulations typify progeroid features.

## 6. Conclusions and Perspectives

Epigenetic code and chromatin status are strictly interconnected and exhibit their effects on cell proliferation and differentiation by regulating the gene expression profile of every single cell. During normal skin homeostasis and tissue renewal, epigenetic mechanisms govern the decision between epidermal SC self-renewal and differentiation toward the fully differentiated keratinocytes.

By regulating the expression of several chromatin remodelers and histone modifying enzymes, the transcription factor p63 is a key player in this process. Furthermore, epigenetic modifications maintain cellular stemness by tightly regulating the expression of the tumor suppressor p16^INK4a^, which orchestrates the cell cycle exit and senescence response. Indeed, epigenetic mechanisms also specify cell senescence and, therefore, delineate the naturally occurring aging process of both epidermal cells as well as dermal fibroblasts. Some of these aging-related epigenetic marks have been found in the skin cells of young individuals, who are affected by premature aging disorders. This finding emphasizes the relevance of PSs for the identification of longevity pathways and potential therapeutic approaches for aging. Most of the progeroid disorders are characterized by genome instability and high cancer susceptibility. Therefore, investigations on PSs will hopefully lead to the identification of novel therapeutic approaches relevant to aging and carcinogenesis. The easy accessibility of the skin makes this tissue the favorite model system to investigate the molecular alterations and dysfunctions of aging-associated disorders.

Since epigenetic modifications are reversible, several studies point to the potential of epigenetic therapies as a tool to reprogram cell fate. Epigenetic therapies are successfully applied for the treatment of some hematological malignancies. However, a deeper knowledge and further studies on skin epigenetic mechanisms are required to achieve the realization of epigenetic therapeutic approaches directed to alleviate the signs of aging in the human population.
